# Genetic variations affecting ACE2 protein stability in minority populations

**DOI:** 10.3389/fmed.2022.1002187

**Published:** 2022-10-31

**Authors:** Vidhyanand Mahase, Adebiyi Sobitan, Raina Rhoades, Fuquan Zhang, Ancha Baranova, Mark Johnson, Abiodun Otolorin, Qiyi Tang, Shaolei Teng

**Affiliations:** ^1^Department of Biology, Howard University, Washington, DC, United States; ^2^Department of Psychiatry, The Affiliated Brain Hospital of Nanjing Medical University, Nanjing, China; ^3^School of Systems Biology, George Mason University, Manassas, VA, United States; ^4^Research Centre for Medical Genetics, Moscow, Russia; ^5^Department of Community and Family Medicine, Howard University, Washington, DC, United States; ^6^Department of Microbiology, Howard University College of Medicine, Washington, DC, United States

**Keywords:** ACE2, SARS-CoV-1-S, SARS-CoV-2-S, genetic variations, protein stability

## Abstract

While worldwide efforts for improving COVID-19 vaccines are currently considered a top priority, the role of the genetic variants responsible for virus receptor protein stability is less studied. Angiotensin-converting enzyme-2 is the primary target of the SARS-CoV-1/SARS-CoV-2 spike (S) glycoprotein, enabling entry into the human body. Here, we applied computational saturation mutagenesis approaches to determine the folding energy caused by all possible mutations in ACE2 proteins within ACE2 - SARS-CoV-1-S/ACE2 - SARS-CoV-2-S complexes. We observed ACE2 mutations at residue D350 causing the most stabilizing effects on the protein. In addition, we identified ACE2 genetic variations in African Americans (rs73635825, rs766996587, and rs780574871), Latino Americans (rs924799658), and both groups (rs4646116 and rs138390800) affecting stability in the ACE2 - SARS-CoV-2-S complex. The findings in this study may aid in targeting the design of stable neutralizing peptides for treating minority patients.

## Introduction

Following the discovery of ACE2 as the SARS-CoV-2 Spike (S) receptor, a substantial number of articles have appeared attempting to characterize the pathology of the infection and help with the creation of therapies (1). As a cellular receptor for SARS viruses, ACE2 protein acts as a port of its entry, allowing the coronavirus to invade and begin replicating. Notably, the S protein of SARS-CoV-2 binds to ACE2 more strongly than SARS-CoV-1 (1).

SARS-CoV-2 replication can be widespread; it is not confined to the lungs, but it can also affect the heart, intestines, blood vessels, muscles, and brain, leading to a myriad of post-COVID sequelae and comorbidities (2–5). Accordingly, many medical conditions aggravating the course of SARS-CoV-2 are characterized by changes in mRNA levels or the membrane abundance of ACE2. Key risk factors for COVID-19 include obesity, diabetes, cardiovascular diseases, and smoking (6–9). In particular, upregulation of ACE2 is a pathophysiological feature of diabetes and hyperglycemic states (10), obesity (11), smoking and air pollution (12, 13). On the other hand, asthma patients show lower expression of ACE2, and it has been reported that asthma exerts a protective effect against SARS-CoV-2 infection as well as severe COVID-19 outcomes (14, 15).

In addition, through the renin-angiotensin system (RAS) (16), ACE2 plays a crucial role in the development of hypertension, atherosclerotic plaque development, and chronic kidney disease (17). Under physiological conditions, the ACE enzyme alters angiotensin I and converts it into angiotensin II, which causes blood vessels to constrict. The tightening of the blood vessels leads to an increase in blood pressure. Recently, ACE2 was identified in pericytes of the heart (17). There are more ACE2 receptors on the surface of cells in the heart muscle in people with established cardiovascular disease than in those without disease (17, 18).

The ACE2 receptor has two functional domains: the N-terminal peptidase M2 domain and the C-terminal collectrin domain. The homodimer side chain domain (residues 19-768) includes the following regions: peptidase domain (PD): 19-65, C-terminal collectrin-like domain (CLD): 616-768, and N-terminal amino acid residues (17 to 537) (19). In both ACE2-SARS-CoV-1/2 complexes, the peptidase and N-terminal domains interact with the spike glycoprotein of SARS-CoV-1 and SARS-CoV-2; however, the N-terminal residues (303-537) are the binding site associated with the spike glycoprotein (20).

For a majority of human proteins, the levels of ACE2 depend on underlying variation in human genomes. Moreover, ACE2 protein species may differ in their amino acid sequences, encoded by genetic alleles unevenly distributed in patients of various ethnic backgrounds. Now that the 1,000 Genomes (16) and the gnomAD (21) projects are completed, common ACE2 variants have already been identified. However, detailed analyses of ACE2 sequences in various African/Latino American populations are still warranted. Several coding variants of ACE2 in humans have been associated with health conditions such as cardiovascular disorders, hypertension, and diabetes. GWASs have described the changes in the coronavirus spike protein and host ACE2 receptor by associating risk variants common contributing to comorbid states (22). A majority of uncovered genetic variation, however, is not directly responsible for a diseased state but serves as biomarkers instead, by pinpointing a set of disease-related loci on the human genome map, by their genetic associations. Occasionally, a variant may be used to seek out and isolate the disease-causing gene directly (23).

Due to widespread inconsistencies in mapping and quantifying underlying genetic variation in world populations, the role of *ACE2* gene variation in differential susceptibility to COVID-19 infections cannot be ruled out. Underprivileged populations, particularly African and Latino Americans, are nearly three times more likely to be hospitalized with COVID-19 than white Americans (24). On the one hand, in these populations, both comorbidities and socioeconomic factors contribute to the higher COVID-19-attributed mortality (25). On the other hand, the African American population shows increased molecular expression of ACE2 (26). Moreover, in 2020, the infection rate for African Americans was 62 per 10,000 compared with 23 per 10,000 for Caucasian Americans (27). The ethnic differences in ACE2 expression may underline the higher infectiveness of SARS-CoV-2 among African Americans (28). Similar to African Americans, Hispanics/Latinos are 1.6 times more likely to develop COVID-19 than their non-Hispanic white counterparts, 3.3 times more likely to be hospitalized from COVID-19, and 2.2 times more likely to die from COVID-19 (28). The Latino population also has a higher infection rate (73 per 10,000) than African Americans (27).

Our study utilizes computational approaches to investigate the effects of every possible ACE2 missense mutation on protein stability. First, we applied structure-based energy calculations to measure the total effects of ACE2 mutations on the protein stability of ACE2-S complexes. Second, we identified key genetic mutations altering ACE2 stability in African/Latino American populations. Finally, we discussed the target residues that need future experimental validation to design neutralizing peptides against SARS-CoV-2.

## Materials and methods

### Structure preparation

The structures ACE2 - SARS-CoV-1-S (PDB ID: 2AJF) and ACE2 - SARS-CoV-2-S (PDB ID: 6LZG) were retrieved from the Protein Data Bank (29). PyMol was employed to visualize structural models and determine structural similarity in both complexes (30). Genetic variations were filtered specifically for the ACE2 gene in African/Latino Americans from gnomAD v4.3 (21). TM-align was used to maximize residue alignment based on structural similarity (30).

### Free energy calculations

FoldX was utilized for all energy calculations, computing the change in the free folding free energy ΔΔG (the difference between the free energy of the mutant and wild-type protein). Each residue position underwent mutagenesis by using the FoldX algorithm (31). The initial step used the “RepairPdb” command to repair the wild-type protein structure, which functions by mutating certain residues to itself to lessen the total free energy of the protein structure. The “Build Model” command (31) is used to calculate the folding energy changes upon mutation. We performed computational saturation mutagenesis on the ACE2 protein and measured the effects of mutations on the protein stability of the S-ACE2 complex. 23,860 mutations were analyzed in the ACE2 chains in both ACE2-Spike protein complexes (PDB: 6LZG and 2AJF, respectively). In addition, we also analyzed the effects of these mutations in ACE2 chains alone by removing the spike chains from the protein complexes.

A value of ΔΔG > 0 shows that the mutation is destabilizing, while a ΔΔG < 0 is a stabilizing mutation. This algorithm has been used in many studies involving protein stabilization experiments (32). Prior to performing all energy analyses, the “RepairPDB” function in FoldX was used to correct bad angles, van der Waals energy clashes, and rotation assignments. For free folding energy calculation, the unfolded protein is considered as two parts, which include a three-residue segment with the mutation in the center and then all other residues. The values of protein stability between the mutant (MUT) and the wild-type structures were computed based on the folding energy change (ΔΔG) by using the following equations:


(1) $$\Delta G_{\left(folding\right)}=G_{\left(folded\right)}-G_{\left(unfolded\right)}$$

(2) $$\Delta \Delta G_{\left(stability\right)}=\Delta G_{\left(folding\right)}MUT-\Delta G_{\left(folding\right)}WT$$

The computational predictions of protein stability changes upon mutations from mCSM (Cutoff Scanning Matrix) (33) and SAAFEC-SEQ (Sequence-based Single Amino Acid Folding Free Energy Changes) (34) were used to compare with the results from FoldX. The R graphical packages were used to generate heatmaps, line graphs, and boxplots (35).

### Sequence-based analysis

Screening for non-acceptable polymorphisms (SNAP), a neural network-based tool used for the evaluation of functional effects of single amino acid substitutions in proteins, was used to predict whether a mutation is likely to alter protein function (23). The ACE2 protein sequences of PDB structures were used as the inputs of SNAP. R-programming was used to represent data by creating suggestable relationships by boxplots to compare the SNAP values.

## Results

### Structural alignment of ACE2 structures in complexes

**FIGURE 1:**
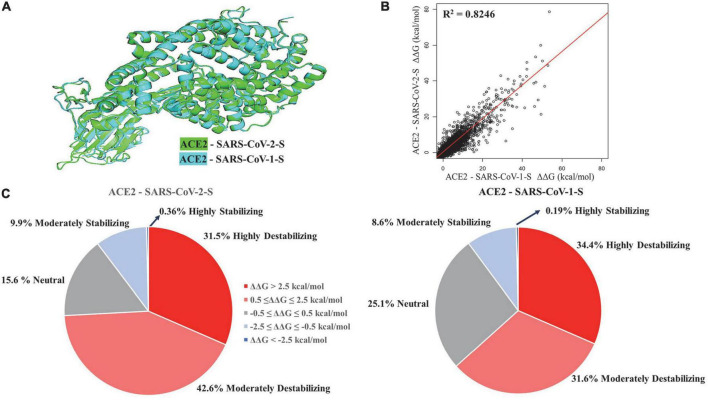
Structural alignment **(A)** and regression analysis **(B)** of ACE2 - SARS-CoV-2-S and ACE2 - SARS-CoV-1-S complexes. Pie charts **(C)** displaying the distribution of effects of mutations on protein stability effects of missense mutations.

We performed structural alignment of ACE2 chains in ACE2 - SARS-CoV-1-S/ACE2 - SARS-CoV-2-S complexes (Figure 1A). The root-mean-square deviation (RMSD) was 0.53, which indicates a great similarity of ACE2 conformations between these two complexes. The ΔΔG values of ACE2 mutations in both complexes were compared (Figure 1B), and the stability effects of mutations on both complexes were correlated (*R*^2^ = 0.8246). Structural alignment and correlation analysis revealed great similarity in ACE2 mutation effects between ACE2 - SARS-CoV-2-S and ACE2 - SARS-CoV-1-S complexes. In addition, we removed the Spike chains in complexes and calculated the ΔΔG of mutations on ACE2 chains alone. As shown in Supplementary Figure 1, the stability effects of mutations on ACE2 monomers are highly correlated with those in the complexes (*R*^2^ = 0.9631 for ACE2 - SARS-CoV-2-S and *R*^2^ = 0.9633 for ACE2 - SARS-CoV-1-S). The top six residue positions that strongly stabilize or destabilize ACE2 protein are listed in Table 1 based on the mean ΔΔG values in complexes. These residues also have common top values mean ΔΔG in ACE2 alone.

**TABLE 1:** Comparison of target mutations among different computational tools on ACE2 – SARS-CoV-1-S/ACE2 – SARS-CoV-2-S ΔΔG_Mean_ (kcal/mol).

Residue position	FoldX			mCSM		SAAFEC-SEQ	
			
	Score		Effect	Score	Effect	Score	Effect
	
	ACE2 - SARS-CoV-2-S	ACE2 Mutation					
G561	24.80	25.46	D	1.23	D	1.57	D
G486	23.94	24.35	D	0.94	D	1.49	D
G268	17.15	17.60	D	1.08	D	1.15	D
G405	17.00	11.48	D	1.22	D	1.42	D
G399	14.80	14.56	D	0.93	D	1.36	D
G173	14.62	14.63	D	1.33	D	1.19	D
D350	–1.52	–1.28	S	0.001	D	–0.51	S
D382	–1.08	–0.12	S	–1.02	S	–0.5	S
G66	–0.92	–0.87	S	–0.98	S	–0.48	S
S105	–0.84	–0.86	S	–0.52	S	–0.58	S
S47	–0.84	–0.76	S	–0.77	S	–0.75	S
S167	–0.79	–0.60	S	–0.63	S	–0.46	S

	**ACE2 - SARS-CoV-1-S**	**ACE2 Mutation**					

G561	29.23	29.38	D	1.23	D	1.57	D
G486	20.73	21.08	D	0.94	D	1.49	D
G173	17.61	17.20	D	1.33	D	1.48	D
G405	16.03	15.74	D	1.22	D	1.42	D
G399	13.82	13.33	D	0.93	D	1.36	D
G268	14.20	14.65	D	1.08	D	1.15	D
D350	–1.58	–1.38	S	0.19	D	–0.60	S
E375	–1.46	–1.39	S	–0.87	S	–0.70	S
D543	–1.43	–1.33	S	–0.15	S	–0.10	S
T324	–1.29	–1.29	S	–0.73	S	–0.49	S
E430	–1.29	–1.03	S	–0.24	S	–0.17	S
S43	–1.10	–1.06	S	–0.89	S	–0.52	S

D, destabilization; S, stabilization.

### Effects of ACE2 mutations on protein stability

The percentage of mutations in both complexes was grouped based on highly destabilizing, moderately destabilizing, neutral, moderately stabilizing, and highly stabilizing (Figure 1C). In the ACE2-SARS-CoV-2-S complex, 31.5% of the mutations displayed high destabilization (ΔΔG > 2.5 kcal/mol), while 42.6% of the mutations showed moderate destabilization (0.5 ≤ ΔΔG ≤ 2.5 kcal/mol). 15.6% of the mutations were neutral (−0.5 ≤ ΔΔG ≤ 0.5 kcal/mol), 9.9% of mutations can moderately stabilize ACE2 (−2.5 ≤ ΔΔG ≤ −0.5 kcal/mol), and 0.36% of the mutations were highly stabilizing (ΔΔG < −2.5 kcal/mol). In the ACE2-SARS-CoV-1-S complex, 34.4% of the mutations displayed high destabilization (ΔΔG > 2.5 kcal/mol), while 31.6% of the mutations showed moderate destabilization (0.5 ≤ ΔΔG ≤ 2.5 kcal/mol). 25.1% of the mutations were neutral (−0.5 ≤ ΔΔG ≤ 0.5 kcal/mol), 8.6% of the mutations had a moderate stabilizing effect (−2.5 ≤ ΔΔG ≤ −0.5 kcal/mol), and 0.19% of the mutations were highly stabilizing (ΔΔG < −2.5 kcal/mol).

**FIGURE 2:**
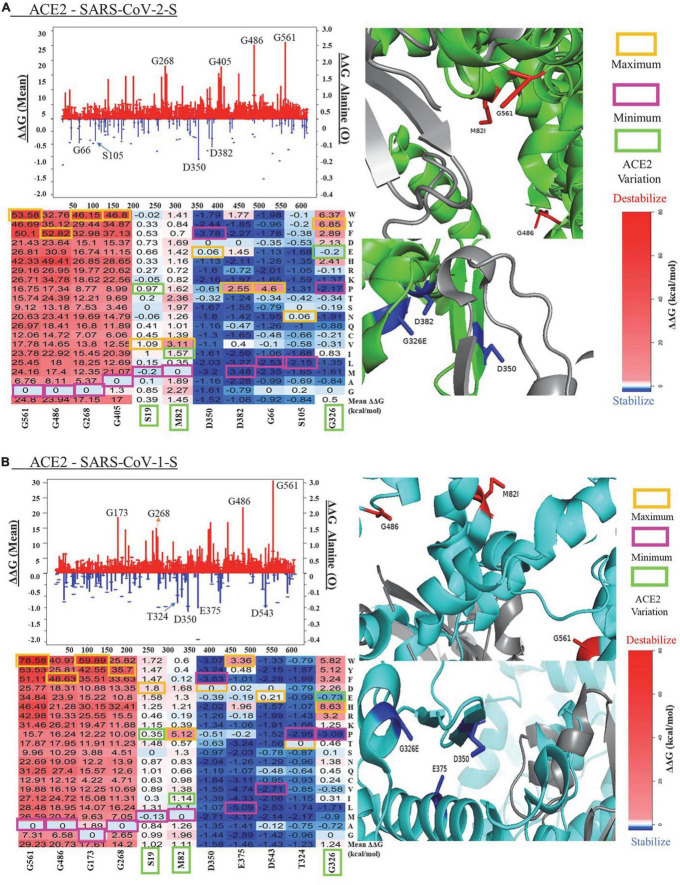
Visualization of key residues affecting protein in panel **(A)** ACE2 - SARS-CoV-2 and **(B)** ACE2 - SARS-CoV-1-S complexes. The lines (mean ΔΔG, kcal/mol) and bubbles (ΔΔG of alanine mutations, kcal/mol) shown the destabilization (red) and stabilization (blue) effects (The scales of y-axis for two effects are different for clear visualization). ACE2 is shown in green or cyan, and the SARS-CoV-2/1 (S) protein is shown in gray. In the heatmaps of key residues, maximum values (orange), minimum (magenta), and the genetic variants (green) are labeled for the substitutions.

The mean values of ΔΔG at each ACE2 residue position and ΔΔG value of substitutions to alanine are shown in the line charts of Figure 2. In the ACE2-SARS-CoV-2-S complex, the mutations in G561 cause the maximum destabilizing effects (mean ΔΔG = 24.80 kcal/mol), while the mutations in D350 have the highest stabilizing effects (mean ΔΔG = −1.52 kcal/mol). A similar pattern of mean ΔΔG was observed in the ACE2 - SARS-CoV-1-S complex, with a range from 29.23 kcal/mol in G561 to -1.58 kcal/mol in D350. The heatmaps of top residues with the maximum destabilizing/stabilization effects of mutations on ACE2 stability and the structural representations of these key residues are shown in Figure 2. Here, we probed for the most destabilizing residues that were common to both complexes. As shown in Figure 2 and Table 1, the mutations at Glycine (G) residue positions G561, G486, G268, G405, and G399 have the maximum destabilizing effects on ACE2 in both complexes. Glycine is the simplest and non-essential amino acid because it has only a hydrogen atom as its side chain. Mutation of G to other larger amino acids will result in adverse conformation variations and destabilize the protein complex, which makes it responsible for over 15% of genetic diseases (36). Its size is often critical in allowing polypeptide chains to make tight turns or to approach one another closely. Residues that replace glycine disturb the helix fold, subjecting chains to additional hydroxylation and glycosylation. Hence, since glycine is crucial in helix formation, substitution at numerous nucleotide sites will result in a clinically evident phenotype (37). Consequently, glycine to glutamic acid substitution can reduce the activity of ACE2 in the central nervous system, thereby affecting the signal transduction between ACE2 in pleural cavities. Interestingly, when G is mutated to tryptophan (W), it causes the highest destabilization energy. A G to W substitution can interact with other aromatic or positively charged residues. W is the largest amino acid and is found to have the highest probability of causing disease [Klein (38)]. In both complexes, the greatest energy instability was seen when G was mutated to W at positions 561, 405, and 399. The greatest unstable energy value was also seen in G561W in both ACE2 - SARS-CoV-1-S/ACE2 - SARS-CoV-2-S (ΔΔG = 78.58 and 53.58 kcal/mol, respectively).

We compared the computational predictions from mCSM and SAAFEC-SEQ to the FoldX results (Table 1). For the six residues with destabilizing effects, all three tools give consistent predictions for the mutation stability effects. FoldX and SAAFEC-SEQ predict that mutations in D350 can stabilize ACE2 protein stability, while mCSM prediction shows that mutations in this residue have neutral effects. For the other five top residues with stabilizing effects, all three tools give negative mean ΔΔG values. The results indicate that the FoldX predication is reliable for identifying the key residues altering protein stability.

### Mutation pathogenic analysis of ACE2

The results from SNAP combine exome and genome data from a variety of algorithms to summarize possible pathogenicity. For this study, amino acid changes were compared to calculated SNAP scores (23). The boxplot functions were used since the generated graphs take up less space and are therefore particularly useful for comparing distributions between several groups or data sets. The SNAP score utilizes various physicochemical features of nsSNPs (non-synonymous single nucleotide polymorphisms) substitutions, as well as evolutionary data.

**FIGURE 3:**
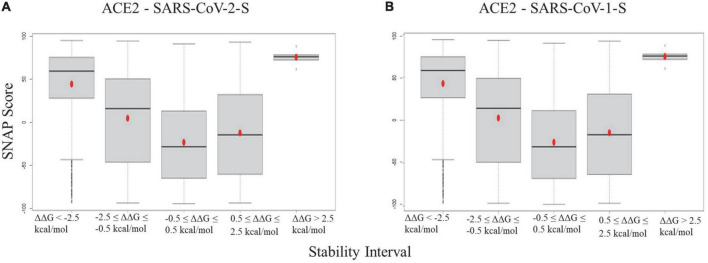
Boxplots for the SNAP scores of ACE2 mutation groups with different folding energy change intervals in panel **(A)** ACE2 - SARS-CoV-2 and **(B)** ACE2 - SARS-CoV-1 complexes.

The boxplots of SNAP scores of mutation groups with different folding energy change (ΔΔG) intervals for ACE2 - SARS-CoV-2-S/ACE2 - SARS-CoV-1-S stability are shown in Figure 3. The SNAP scores were compared versus five categories of stability energy intervals in both complexes, and their means were significantly different (ANOVA test, *p*-value = 0.0036973). The SNAP scores of mutations with strong destabilizing/stabilizing effects (ΔΔG > 2.5 or ΔΔG < -2.5 kcal/mol) were higher than those with moderate effects (0.5 < ΔΔG < 2.5 or no effects (-0.5 ≤ ΔΔG ≤ 0.5 kcal/mol) in both complexes. The results indicate that mutations altering protein stability have damaging effects on ACE2 protein functions.

### Genetic variants affecting protein stability

dbNSFP (39) contains genetic variants from numerous databases (gnomAD, dbSNP, 1000 Genomes, Ensembl, etc.). We collected 231 genetic variants present in gnomAD from dbNSFP v4.3 (Supplementary Table 1). Among these variants, 40 unique missense mutations were observed in African Americans, while 42 unique missense mutations were present in Latino Americans. The selected target genetic variants affecting ACE2 stability in African/Latino Americans are listed in Table 2. rs4646116 (K26R) (more cases are reported in Latino Americans) and rs138390800 (K341R) are found in both ethnic groups, with effects being neutral in both complexes. The variants rs73635825 (S19P), rs146676783 (E37K), and rs766996587 (M82I) identified in African Americans but not Latino Americans can destabilize the ACE2 protein.

**TABLE 2:** Key genetic variants altering ACE2 stability in African/Latino Americans.

Genetic variation		gnomAD allele count*			ΔΔ G (kcal/mol)	
		
dbSNP ID	Mutation	All	AFR	AMR	ACE2 - SARS-CoV-2-S	ACE2 - SARS-CoV-1-S
rs73635825	S19P	46	45	0	0.973	0.350
rs4646116	K26R	728	13	90	0.461	0.418
rs146676783	E37K	6	1	0	0.943	2.857
rs766996587	M82I	2	2	0	1.570	1.136
rs759579097	G326E	1	1	0	–0.195	–0.732
rs924799658	F40 L	3	0	3	0.104	–0.354
rs780574871	E312K	2	2	0	0.276	0.299
rs138390800	K341R	59	52	5	–0.079	0.305

*All, all populations; AFR, African American; AMR, Latino/Admixed American.

## Discussion

There are several reasons why African American and Latino communities are being disproportionately affected by the coronavirus. In particular, these populations are also at higher risk of chronic health problems aggravating the clinical course of COVID-19, including diabetes, heart disease and obesity (6, 40). Here, we concentrated on genetic variants that affect the African/Latino American ethnicities, also facing the greatest socioeconomic and racial disparities (41). Structural studies have reported numerous genetic variations affecting the stability of the ACE2 - SARS-CoV-2-S complex (42). In African Americans, rs766996587 (M82I) and rs73635825 (S19P) appear with an allelic frequency of 1.0 × 10^–5^. rs766996587 represents a methionine to isoleucine change that destabilizes the protein structure and affects the pathogenicity of ACE2-driven viral infections (43). In our study, predicted mutation values show a correlation between ACE2 - SARS-CoV-1-S/ACE2 - SARS-CoV-2-S on respective destabilization (44). rs73635825 (S19P) is a change of a serine to a proline close to the N-end of ACE2 protein. By *in silico* evaluation using PolyPhen-2, rs73635825 has been predicted to have damaging effects, and multiple sources have concluded that the change from serine to proline decreases immune resistance against SARS-CoV-2-S due to a decrease in stability (28). rs73635825 is common in European populations (Allele Frequency = 2.13 × 10^–4^). There have been some published structural and homology modeling studies showing that this variant destabilizes the ACE2 receptor in European populations but not in African Americans (Allele Frequency = 2.1 × 10^–3^) (45). rs146676783 (E37K), a change from glutamic acid to lysine, is detected in African Americans, with an allelic frequency of 3.0 × 10^–5^ appearing in this race (46). E37K contributes to decreased stability in ACE2 - SARS-CoV-2-S (ΔΔG = 0.943 kcal/mol) and in ACE2 - SARS-CoV-1-S (ΔΔG = 2.857 kcal/mol). rs4646116 (K26R), seen predominantly in Latino Americans, destabilizes the ACE2 receptor domain. rs759579097 (G326E), albeit showing a neutral effect on protein stability, increases the binding affinity between ACE2 and SARS-CoV-2-S (47).

We hypothesize that by analyzing the mutant protein stability of both ACE2 - SARS-CoV-1-S/ACE2 - SARS-CoV-2-S complexes, our contribution can aid in predicting future mutations. Increased protein stability can be a significant factor in the study of protein evolution (48). Stabilized proteins can adapt to a broad range of mutations and determine the gradual change of the proteins. Stabilizing mutations offer an advantage to the virus by improving the ratio of correctly folded proteins to decreased protein deficiency inside the cell. Changes in significant amino acids of the ACE2 receptor were projected to decrease or increase SARS-CoV-2-S recognition (49). For example, the hemagglutinin protein of influenza virus has demonstrated that at higher stability, the virulence and infectivity rate are higher (38). Our results show that mutations in residue D350 can increase the stability of the ACE2 receptor in both ACE2 - SARS-CoV-2-S (mean ΔΔG = −1.52 kcal/mol) and ACE2 - SARS-CoV-1-S (mean ΔΔG = −1.58 kcal/mol) complexes. Most COVID-19 vaccines are designed based on the SARS-CoV-2 S protein, and their effectiveness could be influenced by the emergence of new SARS-CoV-2 variants with multiple S mutations. The designed ACE2 could be a potential new therapeutic against COVID-19 (50). Increasing protein stability is an important goal for protein engineering. Target residues can be analyzed and verified by using bioinformatic tools and molecular biology experiments. The replacement of more functional amino acids in these key sites can generate more stable therapeutic peptides. Given the transmission and rapid spread of SARS-CoV-2, it is important to use new computational approaches to design new therapies. Theory and computational systems along with advanced laboratory techniques will increase our understanding of the functional mechanism of SARS-CoV-2 and its human receptor ACE2.

## Conclusion

The excess of publications associated with the SARS-CoV-2-S virus-related disease outbreak reveals the intense effort by researchers to tackle both molecular mechanisms and therapeutic methods useful for treating current and future variants of the coronavirus outbreak. In our research, we showed that mutations in some residues, such as D350, stabilize the ACE2 receptor. Our data indicate that genetic variants, such as rs73635825, rs4646116, rs146676783, and rs766996587, in African/Latino American populations may cause significant decreases in the stability of ACE2. Further research in the areas of antiviral discovery will enhance our understanding of the effects of these mutations in hopes of designing therapeutic peptides capable of disrupting the complexes between the virus and ACE2.

## Data availability statement

The datasets presented in this study can be found in online repositories. The names of the repository/repositories and accession number(s) can be found in the article/Supplementary material.

## Author contributions

VM and ST: conceptualization, software, investigation, data curation, and writing—original draft preparation. VM: methodology and formal analysis. ST, QT, FZ, AB, VM, RR, AO, and MJ: writing—review and editing. QT and ST: supervision. ST: project administration and funding acquisition. All authors contributed to the article and approved the submitted version.

## Supplementary material

Supplementary Figure 1 The regression analysis of ΔΔG (kcal/mol) of mutations in ACE2 chain alone and in complex for ACE2 - SARS-CoV-2-S (left) and ACE2 - SARS-CoV-1-S (right) complexes.

Supplementary Table 1 Summary of ACE2 genetic variants with their gnomAD allele counts and ΔΔG values (kcal/mol).
