# Supplementary figures and images for: Genetic variations affecting ACE2 protein stability in minority populations
Source: Front Med (Lausanne). 2022 Oct 31;9:1002187. doi: 10.3389/fmed.2022.1002187 (PMC9659633; doi:10.3389/fmed.2022.1002187)

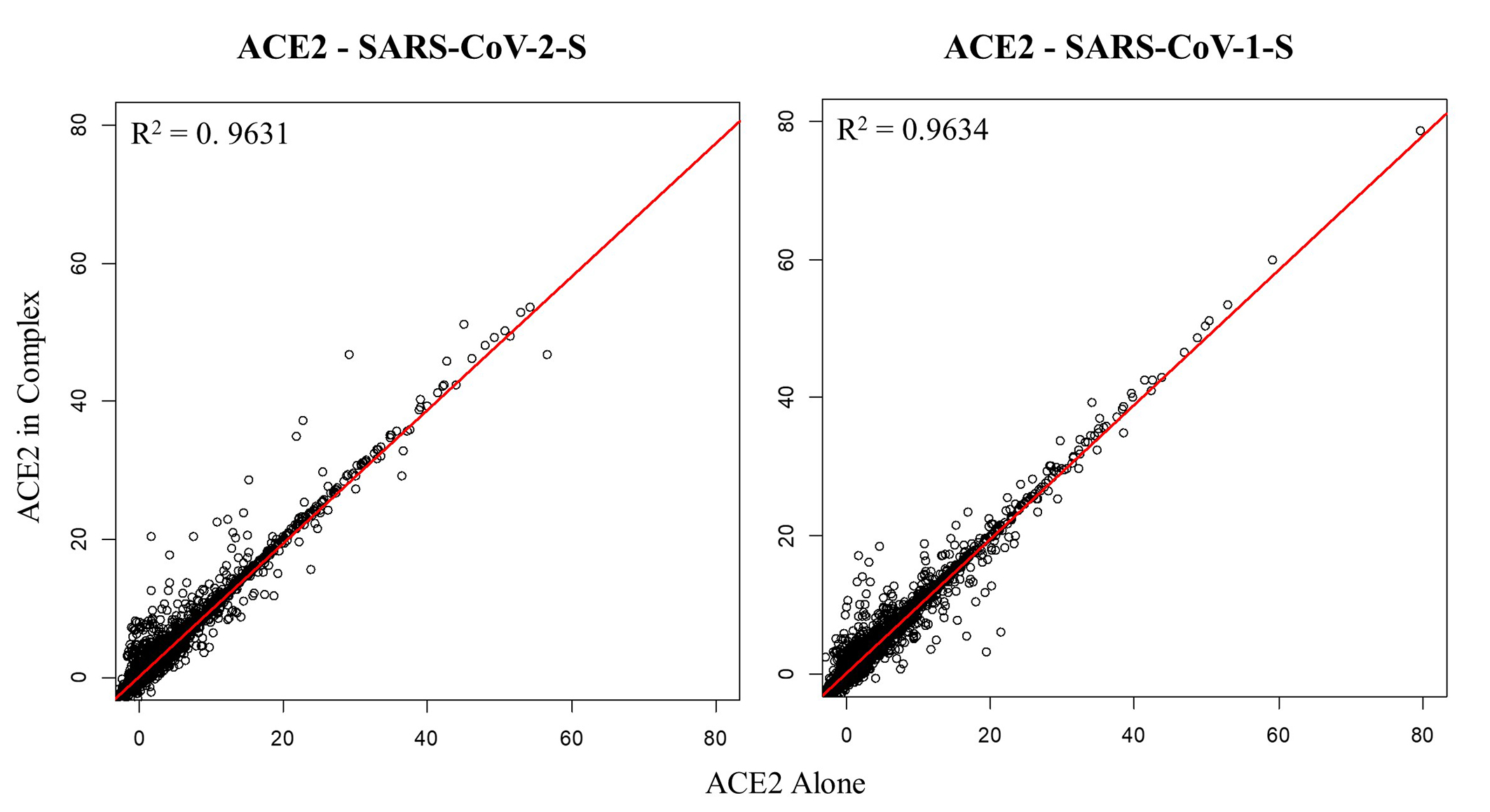

Supplement: Supplementary Figure 1 — The regression analysis of ΔΔG (kcal/mol) of mutations in ACE2 chain alone and in complex for ACE2 - SARS-CoV-2-S (left) and ACE2 - SARS-CoV-1-S (right) complexes. [file Image_1.JPEG]
